# Brain Insulin Resistance and Hippocampal Plasticity: Mechanisms and Biomarkers of Cognitive Decline

**DOI:** 10.3389/fnins.2019.00788

**Published:** 2019-07-31

**Authors:** Matteo Spinelli, Salvatore Fusco, Claudio Grassi

**Affiliations:** ^1^Institute of Human Physiology, Università Cattolica del Sacro Cuore, Rome, Italy; ^2^Fondazione Policlinico Universitario Agostino Gemelli IRCCS, Rome, Italy

**Keywords:** brain insulin resistance, hippocampus, Alzheimer’s disease, synaptic plasticity, adult neurogenesis

## Abstract

In the last decade, much attention has been devoted to the effects of nutrient-related signals on brain development and cognitive functions. A turning point was the discovery that brain areas other than the hypothalamus expressed receptors for hormones related to metabolism. In particular, insulin signaling has been demonstrated to impact on molecular cascades underlying hippocampal plasticity, learning and memory. Here, we summarize the molecular evidence linking alteration of hippocampal insulin sensitivity with changes of both adult neurogenesis and synaptic plasticity. We also review the epidemiological studies and experimental models emphasizing the critical role of brain insulin resistance at the crossroad between metabolic and neurodegenerative disease. Finally, we brief novel findings suggesting how biomarkers of brain insulin resistance, involving the study of brain-derived extracellular vesicles and brain glucose metabolism, may predict the onset and/or the progression of cognitive decline.

## Introduction

Since the discovery of insulin almost a century ago, much efforts have been conducted to study the effects of this hormone on all organs. Insulin was originally shown to act on the brain by stimulating the hypothalamic satiety center and inhibiting the feeding behavior (Debons et al., 1970). For long time the impact of insulin on other brain areas remained unknown because the central nervous system was considered a non-insulin dependent tissue. In the last decades, the discovery of insulin receptor (IR) expression in brain areas involved in functions different from the feeding control, such as learning and memory, has revolutionized this idea and paved the way for the understanding of how the brain is a highly insulin-sensitive organ (Hill et al., 1986; Zhao and Alkon, 2001). Brain plasticity, the capability of this organ to undergo structural and functional changes in response to environmental stimuli, is finely modulated by diet and nutrient-dependent hormones including insulin (Mainardi et al., 2015). Accordingly, alteration of insulin signaling into the central nervous system may accelerate brain aging, affect brain plasticity and promote neurodegeneration (Kullmann et al., 2016).

Here, we review the effects of insulin on hippocampus, a brain area playing a pivotal role in learning and memory and primarily affected in Alzheimer’s disease (AD) (Bartsch and Wulff, 2015). First, we will describe the effects of insulin on both hippocampal synaptic plasticity and hippocampal adult neurogenesis. In addition, we will illustrate the crosstalk between insulin and neurotrophin signaling, the impact of insulin on cognitive function and the role of its signaling on brain aging. Moreover, we will describe how alteration of brain insulin signaling develops (i.e., brain insulin resistance, hereinafter named BIR) and we will summarize the effects of BIR on hippocampal plasticity, learning and memory along with the link between BIR and AD.

Finally, we will discuss novel evidence suggesting that both brain-derived extracellular vesicles and brain glucose metabolism may represent novel biomarkers of BIR able to predict and follow up cognitive decline.

## Insulin and Brain Plasticity

Neurons are high energy-consuming cells. Most energy is spent to generate action and postsynaptic potentials (Howarth et al., 2012), and for the biosynthesis of neurotransmitters (Dienel, 2012). Glucose is the main energy source used by brain cells and its transport across the plasma membrane is mediated by a specific family of membrane proteins known as glucose transporters (GLUTs) (Shepherd and Kahn, 1999). Though numerous GLUT isoforms (1–14) have been identified and characterized, only some of these transporters are expressed in the brain and can be involved in neuronal homeostasis and brain function (Duelli and Kuschinsky, 2001). Specifically, the insulin-independent transporters GLUT1 and GLUT3 mediate glucose uptake into glial and neuronal cells, respectively (Simpson et al., 2007), suggesting that the impact of insulin on synaptic plasticity should be independent of glucose uptake.

Moreover, GLUT2 and GLUT4 expression has been characterized in specific brain areas: GLUT2 is predominantly localized in the hypothalamus that regulates food intake (Eny et al., 2008), whereas GLUT4 has been identified in cerebellum, neocortex, and hippocampus, suggesting a role of GLUT-driven glucose uptake in neuronal activity (Vannucci et al., 1998; Sankar et al., 2002). GLUT4 is also expressed in astrocytes and insulin stimulation promotes both glucose uptake and glycogen accumulation in astrocyte cultures (Heni et al., 2011). However, not much data are available in the literature on the role of insulin on astrocytic functions. GLUT5 expression is less relevant and mainly detected in human and rat brain microglia (Payne et al., 1997). GLUT6 and GLUT13, which have very low affinity to glucose, are also expressed in the brain, but their role in central nervous system has yet to be clarified (Joost and Thorens, 2001). Conversely, GLUT8 has been shown to drive hippocampal neuron proliferation during embryogenesis (Membrez et al., 2006). However, tissue/cell type specific expression of GLUTs in the brain still remains matter of debate. Further, several growth factors have been reported to modulate the insulin pathway, GLUT plasma membrane translocation and glucose uptake by transactivation of the IR downstream effectors (Assefa et al., 2017).

In this section, we brief the insulin cascade effectors and the effects of this hormone on both synaptic plasticity and adult neurogenesis in the hippocampus along with their impact on cognitive functions.

### Insulin Signaling in the Brain

**FIGURE 1:**
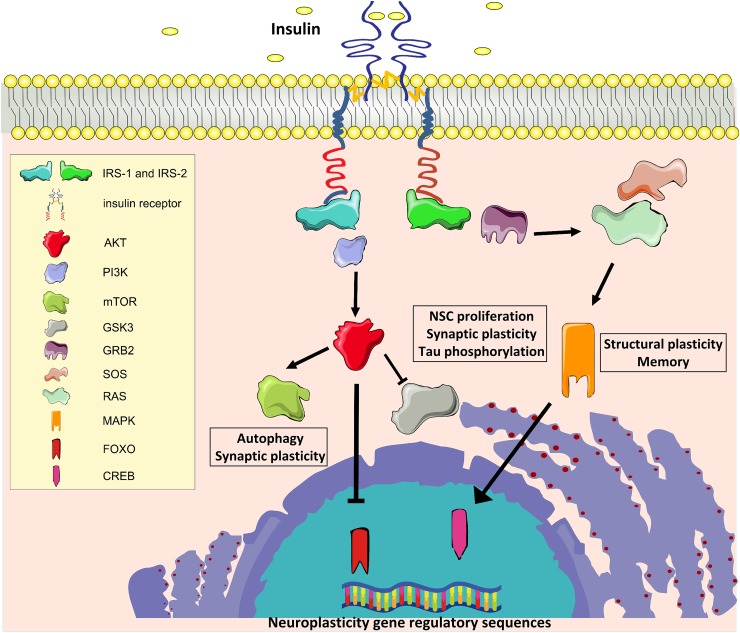
Insulin signaling. IR, Insulin receptor; IRS1 and IRS2, IR substrate 1 and 2; PI3K, phosphoinositide-3-kinase–protein kinase B; AKT, protein kinase B; mTOR, target of rapamycin; GSK3β, glycogen synthase kinase 3 beta; GRB2, growth factor receptor-bound protein 2; SOS, Son of Sevenless; RAS, Rat sarcoma GTPase protein; MAPK, mitogen activated protein kinase; FOXO, forkhead box O transcription factor; CREB, cAMP response element-binding protein.

Insulin and the insulin-like growth factor 1 (IGF-1) exert their biological effects through two tyrosine kinase receptors, the IR and the IGF-1 receptors (IGF-1R), which are closely related and highly distributed throughout the brain (Belfiore et al., 2009). In the mouse, IR is predominantly expressed in the olfactory bulb, hippocampus, neocortex, hypothalamus, and cerebellum, whereas IGF-1R is highly expressed in the hippocampus, neocortex, and thalamus, with lower expression in the hypothalamus, cerebellum, olfactory bulb, midbrain, and brainstem (Bruning et al., 2000; Fernandez and Torres-Aleman, 2012). As reported in other tissues, IRs and IGF-1Rs can heterodimerize in the brain and partially transactivate their signaling (Bailyes et al., 1997). Moreover, IR and IGF-1R share intracellular signaling machinery, and all major components of brain signaling cascades are similar to those present in peripheral tissues, including IR substrate 1 and 2 (IRS1 and IRS2, respectively), the major downstream phosphoinositide-3-kinase–protein kinase B/Akt (PI3K/Akt) pathway, the downstream effectors target of rapamycin (mTOR) and glycogen synthase kinase 3 beta (GSK3β), and the transcription factors cAMP response element-binding protein (CREB) and forkhead box O (FOXO) family (Fernandez and Torres-Aleman, 2012; Figure 1).

Many molecules involved in these signaling cascades have been demonstrated to have key roles in brain functions. Activation of PI3-kinase is required for glutamate receptor insertion at plasma membranes during synaptic plasticity (Man et al., 2003). GSK3β regulates neural progenitor cell proliferation and neuroplasticity, and its activation induces hyper-phosphorylation of tau protein that is considered a major determinant of AD pathogenesis (Salcedo-Tello et al., 2011). Insulin induces phosphorylation of GSK3β on inhibitory serine 9 residue, thus reducing its enzymatic activity. Moreover, mTOR complex 1 (mTORC1) is fundamental for both protein synthesis and autophagy, which are molecular processes involved in the regulation of long-term synaptic plasticity and degradation of misfolded proteins in neurons, respectively (Stoica et al., 2011; Son et al., 2012). Insulin/IGF-1 signaling also stimulates the growth factor receptor-bound protein 2- Son of Sevenless-Rat sarcoma-mitogen activated protein kinase (Grb2-SOS-Ras-MAPK) cascade, which plays a pivotal role in cytoskeletal modifications underlying the dendritic spine reorganization and memory formation (Adams and Sweatt, 2002).

The origin of insulin in the central nervous system is controversial. Insulin crosses the blood-brain barrier (BBB) via an IR-dependent transport operated by vascular endothelium and its concentration increases after meals (Woods et al., 2003). However, insulin can be also synthesized and secreted by neurons and adult neural progenitor cells of the hippocampus (Devaskar et al., 1994; Kuwabara et al., 2011), although no evidence clearly demonstrated that insulin synthesis in the brain is physiologically relevant.

However, as described in the next paragraphs, physiological levels of insulin play a neurotrophic action on both differentiated neurons and neural stem cells (NSCs).

### Insulin, Synaptogenesis and Hippocampal Synaptic Plasticity

Modifications of both activity and number of synapses are the functional and structural substrates, respectively, of brain plasticity underlying learning and memory (Nakahata and Yasuda, 2018). Changes of the synaptic strength, either potentiation or depression, and generation of new dendritic spines are causally related to the acquisition and consolidation of behavioral modifications.

Insulin stimulation of hippocampal neurons induces both presynaptic and postsynaptic effects. Insulin increases basal neurotransmitter release from presynaptic terminals, as revealed by enhanced frequency of miniature excitatory postsynaptic currents (mEPSCs) (Lee et al., 2011). This effect is paralleled by a Rac1-mediated cytoskeleton rearrangement leading to increased density of dendritic spines (Lee et al., 2011). Moreover, insulin promotes synaptic plasticity by modulating long-term potentiation (LTP) or long-term depression (LTD) at hippocampal synapses through a metaplastic mechanism. Indeed, insulin administration reduces the stimulation frequency threshold required for inducing both LTP and LTD (Van Der Heide et al., 2005). Postsynaptic effects are mediated by PI3K activation (Van Der Heide et al., 2005) and increased membrane recruitment of *N*-methyl-D-aspartate receptors (NMDARs) (Skeberdis et al., 2001). Insulin impacts on glutamate receptor activity by multiple mechanisms. It increases NMDAR-mediated currents by enhancing phosphorylation of both NR2A and NR2B subunits (Christie et al., 1999; Liu et al., 1995). Insulin treatment of hippocampal cultures also increases phosphorylation and clathrin-dependent endocytosis of the GluA1 subunit of α-amino-3-hydroxy-5-methyl-4-isoxazolepropionic acid receptors (AMPARs) (Adzovic and Domenici, 2014; Chen et al., 2014). Downregulation of AMPAR activity in excitatory synapse of hippocampal CA1 neurons is fundamental for insulin-induced LTD, which is a key step for memory consolidation and flexibility (Ge et al., 2010). IRs have also been demonstrated to modulate type A γ-aminobutyric acid (GABA) receptor activity by regulating both its membrane localization and expression in inhibitory synapses (Wan et al., 1997).

Furthermore, insulin may impinge on structural features of synapses. For instance, in thalamocortical organotypic slices this hormone stimulates maturation of silent synapses (Plitzko et al., 2001). Moreover, IR substrate p53 (IRSp53) interacts with the postsynaptic protein PSD-95 and enhances dendritic spine formation (Choi et al., 2005). Interestingly, IRS2 knockout mice show lower activation of NR2B subunits (Martin et al., 2012) and decreased LTP at the CA3–CA1 synapses in parallel with higher density of CA1 dendritic spines (Irvine et al., 2011). It is important to underline that these studies did not evaluate dendritic spine morphology, therefore our knowledge of the effects of IRSs manipulation on structural and functional plasticity still remains incomplete. Considering the physical and functional interaction between IR and IGF-1R, it is not surprising that IGF-1 stimulation can promote plasticity in the hippocampus by increasing spine density of CA1 basal dendrites in response to physical exercise (Glasper et al., 2010). Accordingly, IGF-I knockout mice show reduced density of glutamatergic synapses (Trejo et al., 2007). Importantly, IR expression and insulin activity in the brain are not restricted to neurons. Insulin has been demonstrated to influence proliferation and metabolism in insulin sensitive glial cells (Heni et al., 2011). Collectively, all the above mentioned evidence supports the positive effects of insulin on hippocampal synaptic and structural plasticity.

### Insulin and Hippocampal Adult Neurogenesis

Hippocampus is one of the brain areas where newborn neurons are generated throughout adulthood (Braun and Jessberger, 2014). Specifically, adult neurogenesis occurs in the subgranular zone of the hippocampus of all mammals including humans (Eriksson et al., 1998). NSCs populating this neurogenic niche proliferate and differentiate to generate new neurons (Kempermann et al., 2003). A proper balance between NSC proliferation and their differentiation/maturation underlies the maintenance of both the hippocampal stem cell niche and the supply of newborn neurons that integrate into existing circuits thus supporting cognitive functions under physiological conditions and brain repair after injury (Castilla-Ortega et al., 2011). Indeed, a growing number of studies indicates that hippocampal neurogenesis plays a critical role in learning, memory, and its impairment has been associated with cognitive dysfunction in neurodegenerative disorders including AD (van Praag et al., 2002; Taylor et al., 2013).

Insulin is a key trophic factor for brain development and control of neurogenic niches. Indeed, neuroblast exit from quiescence is regulated by insulin/IGF-I pathway activation (Chell and Brand, 2010; Sousa-Nunes et al., 2011). Evidence from *in vitro* and *in vivo* experiments indicate that insulin and IGF-I promote neurogenesis by modulating NSC proliferation, differentiation, and survival (Brooker et al., 2000; Åberg et al., 2003). However, a chronic hyper-activation of insulin/IGF-I signaling cascades can cause premature depletion of the NSC reservoir (Sun, 2006). Thus, insulin may produce either trophic or detrimental effects on neural stem niche based on the timing and the duration of stimulation.

Furthermore, calorie restriction has been clearly demonstrated to reduce plasma levels of both glucose and insulin, in parallel with increasing neurogenesis in the dentate gyrus (Lee et al., 2002) and counteracting the age-related decline of stem cell niche (Park et al., 2013). Nutrient deprivation may impact on NSC compartment by inducing the expression of the brain-derived neurotrophic factor (Bdnf) gene (Maswood et al., 2004). In addition, calorie restriction may preserve the NSC capacity to self-renew and differentiate by cell-autonomous mechanisms involving metabolic sensors such as CREB and the NAD-dependent histone deacetylase Sirtuin 1 (SIRT1). In this regard, CREB is a nutrient-dependent transcription factor regulating genes promoting neuronal differentiation and survival (Lonze et al., 2002; Fusco et al., 2012b). Moreover, SIRT1 is as an epigenetic repressor that modulates adult neurogenesis in the subventricular zone and hippocampus (Saharan et al., 2013). Calorie restriction also induces the expression of SIRT1, which has been shown to functionally impinge on CREB-dependent gene expression, thus highlighting a novel molecular link between nutrient-dependent signaling and brain health (Fusco et al., 2012a). Under metabolic and oxidative stress, SIRT1 inhibits NSC self-renewal and induces their differentiation (Prozorovski et al., 2008; Ma et al., 2014). In summary, SIRT1 and CREB work as metabolic sensors regulating proliferation and self-renewal of NSCs and controlling their reservoir in the hippocampus (Fusco et al., 2016). Conversely, abolishing the expression of genes encoding the insulin-regulated FOXO transcription factors induces hyper-proliferation of neural progenitors and rapid exhaustion of stem cell niche (Renault et al., 2009). Similarly, aberrant stimulation of the nutrient-dependent mTOR pathway causes reduced self-renewal and accelerates NSC differentiation (Magri et al., 2011). Together, this evidence confirms that nutrient related signals control NSC fate under both physiological and pathological conditions.

In addition, it is worth mentioning the close similarity between the intracellular signaling pathways activated by insulin and neurotrophins (Reichardt, 2006). In particular, CREB has been shown to play a critical role in the neurotrophin-triggered effects on neuronal differentiation, survival, and plasticity, and it has been also characterized as metabolic sensor modulated by fasting-related stimuli (Finkbeiner, 2000; Altarejos and Montminy, 2011). Moreover, neurotrophic factors as BDNF, ciliary neurotrophic factor (CNTF), and glial cell-derived neurotrophic factor (GDNF) regulate adult neurogenesis in multiple stages of NSC maturation and their expression is affected by overnutrition and metabolic stress (Lindsay et al., 1994). These evidence emphasizes the role of insulin as growth factor for neural niche especially during the early stages of life.

### Insulin Signaling and Hippocampus-Dependent Cognitive Task

The evidence summarized above suggest that all aspects of hippocampal plasticity (i.e., functional and structural synaptic plasticity, and adult neurogenesis) are strongly sensitive to the modulation of insulin signaling into the brain. According to the key role of hippocampal plasticity in learning and memory, changes of insulin cascade in the hippocampus markedly affects cognitive functions.

Heterozygous knockout mice for IR display lower preference index in the novel object recognition (NOR) test (Nisticò et al., 2012). Accordingly, Zucker rats show lower performance in the Morris Water Maze (MWM) in parallel with impaired insulin sensitivity (Kamal et al., 2013). Moreover, a recent study performed on Goto-Kakizaki (GK) rats, a model of non-obese type 2 diabetes (T2D), display spatial memory impairment in Y-maze task and hippocampal synaptic dysfunction evaluated by LTP (Duarte et al., 2019). Further, GK rats show a reduction of SNAP25 and synaptophysin levels suggesting synapse degeneration (Duarte et al., 2019). In addition, IRSp53 knockout mice show impaired learning and memory when evaluated in both MWM and NOR tests (Kim et al., 2009). However, forebrain-specific IRS2 deficiency improved memory retention in the MWM task (Irvine et al., 2011), suggesting that molecular hubs of insulin signaling may differentially interfere on cognitive behavior.

In agreement with this finding, chronic brain stimulation by 8-week intranasal insulin administration improved memory in humans (Benedict et al., 2004). Moreover, lower values of both glycaemia and glycosylated hemoglobin (HbA1c) are associated with better performance in memory tasks in humans (Kerti et al., 2013). It has been shown that peripheral changes of insulin signaling and sensitivity may affect brain health and function although the results of these studies are controversial. Learning and memory deficits in MWM have been demonstrated in liver-specific IGF1 knockout mice (Trejo et al., 2007). In addition, IGF-I antiserum administration to young rats impairs learning in passive avoidance task (Lupien et al., 2003). Conversely, intraperitoneal injection of insulin impairs retention and spatial working memory in a dose-dependent manner (Kopf and Baratti, 1999; Akanmu et al., 2009). It seems that the negative impact of peripheral insulin administration on cognitive functions may be due to the lowering of blood glucose levels, as indicated by the positive effect of the simultaneous glucose infusion (Kopf et al., 1998). In line with this hypothesis, peripheral administration of insulin increases verbal memory and attention in healthy subjects under euglycemic conditions (Kern et al., 2001).

To avoid the side effects of systemic insulin administration, Park et al. (2000) studied the impact of intracerebroventricular injection of insulin in rats and found that insulin improved cognitive performance in passive avoidance test. Accordingly, intrahippocampal injection of insulin into the CA1 region has been reported to enhance memory of rats in passive avoidance test (Babri et al., 2007). More importantly, the effects of intrahippocampal injection of insulin on cognitive functions seem to be related to its dose. High doses of insulin significantly ameliorate spatial learning and memory in the MWM test, whereas low doses reduce cognitive performance (Moosavi et al., 2006). It has been hypothesized that the negative effects of insulin on spatial memory may be dependent on either upregulation of GABA_A_ receptors or downregulation of AMPA receptors upon insulin treatment (Moosavi et al., 2006). Finally, McNay et al. (2010) demonstrated that endogenous intrahippocampal insulin signaling was required for memory processing. These authors showed that acute injection of insulin into the hippocampus at physiological doses enhanced spatial memory via a PI3K-dependent mechanism (McNay et al., 2010). Collectively, the results obtained in humans and rodents suggest that insulin is fundamental for both memory formation and retention.

## Effects of Brain Insulin Resistance on Hippocampus- Dependent Functions

Data reviewed in the previous paragraphs support the view that changes of either insulin signaling or insulin sensitivity in the hippocampus may alter molecular pathways involved in synaptic plasticity and adult neurogenesis, thereby leading to reduced “mindspan” (the maintenance of mental abilities throughout life) and increased risk of neurodegeneration (Kodl and Seaquist, 2008). Accordingly, while calorie restriction furthers neuronal survival and improves cognitive function (Fusco and Pani, 2013), the excess of nutrients harms the brain health and accelerates cognitive decline (Elias et al., 2012; Sellbom and Gunstad, 2012). Nutrient excess causes hyper-activation of insulin signaling in all tissues expressing IR, leading to the desensitization of IR-dependent molecular cascades. BIR decreases the ability of brain cells to respond to insulin and abolishes both metabolic and cognitive effects of this hormone (Kullmann et al., 2016). Specifically, this deficiency could be caused by lower expression of IR or poor activation of insulin signaling. IR downstream effectors may become insensitive to the insulin stimulation, resulting in inability of brain cells to respond to the hormone and leading to impairment of brain plasticity. In the Western world, the incidence of metabolic disorders, including insulin resistance, obesity and T2D, is increasing at alarming rates in parallel with the prevalence of cognitive decline (Cukierman-Yaffee, 2009). Obesity and inflammation affect the insulin transport to the brain (Ketterer et al., 2011) and low expression of IR has been reported in patients with T2D (Kullmann et al., 2016). However, patients with T2DM and/or obesity showed decreased insulin levels in the cerebrospinal fluid despite higher levels of this hormone in their plasma (Heni et al., 2014). In the following paragraphs, we will summarize the mechanisms underlying the detrimental effects of BIR on hippocampal plasticity and cognition, and the epidemiological and experimental evidence supporting a link between BIR and AD.

### Alterations of Hippocampal Plasticity in BIR Models

High-fat diet (HFD) is a well-established animal model of metabolic disorders (Wong et al., 2016). HFD induces obesity by compromising β-cell functions, promoting hyper-glycaemia, whole-body insulin resistance, and dyslipidemia, and increasing free fatty acids in the blood. Many studies have investigated the structural and functional changes of neuroplasticity in experimental models of insulin resistance (Fadel and Reagan, 2016).

More specifically, HFD produces detrimental effects on brain functions including decreased neurogenesis in the dentate gyrus (Lindqvist et al., 2006), alteration of BBB integrity (Freeman and Granholm, 2012) and changes in both spine density and synapse formation (Stranahan et al., 2008a). HFD also impairs insulin signaling in the hippocampus and reduces the expression of synaptic proteins PSD-95 and synaptopodin (Arnold et al., 2014). However, the most significant effects occur on activity-dependent synaptic plasticity. Indeed, Zucker rats show impairment in LTP at CA3–CA1 synapses in parallel with loss of insulin sensitivity (Kamal et al., 2013). Moreover, IR heterozygous knockout mice display normal levels of both basal synaptic transmission and LTP that, however, fails to be consolidated due to reduced Akt activation (Nisticò et al., 2012).

Obesity and T2D have been demonstrated to induce hippocampal insulin resistance through different metabolic changes including alteration of hypothalamic-pituitary-adrenal (HPA) axis leading to elevated levels of glucocorticoids (Plotsky et al., 1992). Accordingly, glucocorticoids stimulation inhibits translocation of GLUT4 to the plasma membrane in the rat hippocampus (Piroli et al., 2007). Moreover, Stranahan et al. showed that restoring physiological levels of glucocorticoids in insulin resistant db/db mice rescued the impairment of hippocampal synaptic plasticity (Stranahan et al., 2008b). A different model of BIR is obtained by intracerebral injection of streptozotocin, which impairs cognitive function by reducing the activity of the neuroprotective protein SIRT1 (Du et al., 2014). As mentioned before, SIRT1 cooperates with the transcription factor CREB promoting the CREB-dependent expression of the neuroplasticity-related gene Bdnf (Jeong et al., 2012). To better clarify the functional role of hippocampal insulin resistance, Grillo et al. (2015) silenced the expression of IR in the hippocampus by injecting lentiviral particles harboring IR antisense sequence. This experimental model showed deficits in hippocampal synaptic transmission and spatial learning, in parallel with downregulation of NMDA subunit GluN2B expression and lower phosphorylation of AMPA subunit GluA1, without altering peripheral metabolic parameters (i.e., body weight, adiposity, and glucose homeostasis) (Grillo et al., 2015).

Recently, we described a novel link between BIR and altered glutamate receptor function underlying the HFD-dependent impairment of hippocampal synaptic plasticity (Spinelli et al., 2017). In particular, we found that HFD induced accumulation of palmitic acid and increased FOXO3a-dependent expression of palmitoyl-transferase zDHHC3 leading to GluA1 hyper-palmitoylation in the hippocampus. Accordingly, *in vitro* stimulation of hippocampal neurons with a cocktail of insulin and palmitic acid replicated the *in vivo* molecular changes, inhibiting the GluA1 localization at the synaptic membrane and AMPA currents at glutamatergic synapses. Finally, either silencing of zDHHC3 or overexpression of the palmitoylation-deficient GluA1 mutant in the hippocampus abolished the insulin resistance-dependent impairment of synaptic plasticity (Spinelli et al., 2017). Of course, aberrant palmitoylation of other zDHHC3 targets (e.g., GABA_A_ RƔ2) may contribute to the detrimental effects of HFD on hippocampus-dependent learning and memory. However, our study adds a new layer to the hippocampal synaptic plasticity regulation by insulin signaling deterioration and proposes a novel molecular mechanism potentially linking BIR and cognitive decline. Other mechanisms underlying fatty acid-driven learning deficits involve cholesterol dysmetabolism, oxidative stress, endothelial dysfunctions, and neurotrophin depletion. Mice fed with HFD show higher levels of reactive oxygen species (ROS), superoxide, and peroxynitrite into the brain, leading to lower level of brain-derived neurotrophic factor (BDNF) and impaired cognition performance evaluated by spatial task (Wu et al., 2004). Moreover, epidemiological studies showed that diets enriched in cholesterol (HCD) were associated with poor cognitive performance in humans (Requejo et al., 2003). HCD diet also induced impairment of spatial and working memory due to microglial activation and alteration of the BBB integrity in rats (Chen et al., 2018). Interestingly, feeding obese rodents with HFD inhibited the transport through the BBB of neuroendocrine molecules, such as ghrelin and leptin, which promote synaptic plasticity and cognitive functions (Banks et al., 2008; Kanoski et al., 2013; Mainardi et al., 2017). Finally, HFD has been shown to induce activation of microglia and astrocytes, and increase of pro-inflammatory cytokines/mediators such as cyclooxygenase 2, TNF-α, IL-1-β, and IL-6 in the hippocampus of mice (Thirumangalakudi et al., 2008; Duffy et al., 2019).

In summary, metabolic diseases affecting insulin signaling may impair the synaptic function through a plethora of molecular mechanisms targeting neurons, astrocytes, endothelial or inflammatory cells.

### Cognitive Impairment in BIR Models

Epidemiological evidence indicate that metabolic alterations occurring in T2D, such as hyper-glycaemia and hyper-insulinaemia, positively correlate with cognitive impairment and diabetic patients exhibit higher susceptibility to develop dementia (Cukierman-Yaffee, 2009). Dysregulation of glucose homeostasis increases the risk of dementia in both diabetic and non-diabetic patients (Crane et al., 2013) and is associated with reduced hippocampal volume and cognitive decline (Kerti et al., 2013). Furthermore, longitudinal studies demonstrated that also Type 1 diabetes (T1D) patients were affected by mild-severe cognitive impairment related to the age of onset of the disease and the microvascular complications (Moheet et al., 2015; Nunley et al., 2015). Insulin administration is crucial to promote glucose homeostasis in these patients and to reduce the vascular complications but it increases the risk for hypoglycemic episodes, which negatively impact on cognitive functions (Desrocher and Rovet, 2004). However, the role of hypo- or hyper-insulinemia in T1D-related cognitive alterations has still to be clarified.

Numerous clinical studies revealed worse cognitive performance and earlier age incidence of all-cause dementia in subjects with T2D (Davis et al., 2017; Callisaya et al., 2019). Accordingly, meta-analysis studies showed that in diabetic patients the risk for all types of dementia is increased by 60–73% (Gudala et al., 2013; Chatterjee et al., 2016).

However, alteration of brain insulin signaling may negatively impact on brain function also in the absence of T2D and before the onset of obesity. Several studies have demonstrated deficits in hippocampal-dependent learning and spatial memory associated with Western diet intake (Molteni et al., 2002; Kanoski and Davidson, 2010). Interestingly, when Kanoski and Davidson (2010) investigated both hippocampus-dependent and hippocampus-independent memory retention ability after different Western diet treatments, they found that only spatial memory impairments occurred after short-term consumption. This suggests that hippocampus is a brain area very sensitive to metabolic stress, and memory impairment may arise before the development of diet-induced metabolic alterations in peripheral tissues. Accordingly, few days of HFD regimen were sufficient to cause cognitive impairment in rats evaluated with MWM test (Murray et al., 2009). High caloric intake also affected hippocampus-dependent non-spatial learning and memory tasks and these results were related to changes of the BBB integrity. Specifically, high energy diet consumption reduced the expression of tight junction proteins selectively causing increased blood-to-brain permeability in the hippocampus (Kanoski and Davidson, 2010). The observed learning and memory deficits are strikingly similar to the poor task performance and cognitive impairment observed in patients with mild or severe metabolic derangements, which strengthens the hypothesis that hippocampal insulin resistance is a key mediator of diet-dependent cognitive alterations. Therefore, cognitive dysfunction related to HFD or obesity in otherwise healthy individuals may be due to decreased insulin signaling and development of BIR in the hippocampus (McNay et al., 2010).

Nevertheless, peripheral insulin resistance and diet-induced obesity are correlated with some other changes that may cause neurocognitive dysfunction. Indeed, they induce systemic and central inflammation with high levels of circulating pro-inflammatory interleukins that have been linked to impaired executive function (Trollor et al., 2012). Obesity also alters HPA axis causing enhanced secretion of glucocorticoids, which have been associated with reduced hippocampal volume, memory impairment and mood alterations (MacQueen and Frodl, 2011). Moreover, mice specifically lacking the IR into the brain (NIRKO mice) display changes in dopamine turnover associated with anxiety and depressive-like behaviors (Kleinridders et al., 2015). In addition, diet-induced microbiota dysbiosis can impact on the gut-brain axis, thus promoting insulin resistance and cognitive impairment (Daulatzai, 2014). Finally, HFD exposure during early stages of life is associated with impaired learning and spatial memory (Boitard et al., 2012), suggesting that alteration of insulin signaling may negatively influence cognitive function at each stage of life.

### Brain Insulin Resistance, Brain Aging and Neurodegenerative Diseases

While diabetes is known to increase the risk for dementia, the underlying mechanisms linking insulin resistance, T2D and AD are poorly understood. Undoubtedly, micro- and macro-vascular complications of T2D may increase the risk of cerebrovascular disease, cognitive impairment and vascular dementia (Gorelick et al., 2011). Moreover, white matter disease, alteration of the BBB and neuro-inflammation may play a pathophysiologic role (Hsu and Kanoski, 2014). However, hyper-insulinaemia promotes the formation of advanced glucose end products and ROS causing neurotoxicity and brain damage (Brownlee, 2001). Despite insulin exerts a neurotrophic role at moderate concentrations, higher levels of the hormone may be associated with increased deposition of amyloid-β (Aβ) in the brain due to competition for their common and main clearance mechanism, the insulin-degrading enzyme (Farris et al., 2003). In this regard, AD has been defined a form of type 3 diabetes, based on the evidence of BIR development in the AD brain (Steen et al., 2005; Bedse et al., 2015; Sposato et al., 2019). The insulin synthesis decreases during aging and AD progression in brain areas such as frontal cortex, hippocampus, and hypothalamus (Frolich et al., 1998). In addition, Aβ inhibits insulin expression in astrocytes (Pitt et al., 2017). Together, these studies indicate a crosstalk between brain insulin signaling alteration and Aβ accumulation in neurodegenerative diseases. Accordingly, experimental data obtained from neuroimaging and biomarker studies revealed that T2D patients showed alterations of both brain glucose metabolism and cerebrospinal fluid including phosphorylated tau, which are reminiscent of changes observed in AD (Baker et al., 2011; Moran et al., 2015). In addition, analysis of AD postmortem brains revealed insulin signaling alterations in hippocampal tissues resembling the biochemical features of insulin resistance in parallel with histopathological hallmarks of neurodegeneration (Talbot et al., 2012; Tramutola et al., 2015). Moreover, tau is hyper-phosphorylated in the brain of NIRKO mice (Schubert et al., 2004) and BIR has been associated with tau pathology in AD human brains (Yarchoan and Arnold, 2014).

Interestingly, T2D and AD also share several metabolic derangements promoting brain aging. AD patients show hyper-insulinaemia and decreased peripheral insulin sensitivity (Craft et al., 1996), whereas insulin levels in cerebrospinal fluid are reduced (Craft et al., 1998). Accordingly, sustained peripheral hyper-insulinaemia can reduce the transport of insulin into the brain due to the lower expression of IR at the BBB (Schwartz et al., 1990). Brain insulin uptake is also impaired in both aging and AD independently by T2D (Frolich et al., 1998). Recent evidence suggests that insulin may influence Aβ deposition and AD-dependent impairment of both synaptic plasticity and memory formation (Cholerton et al., 2013). Intranasal insulin administration has been demonstrated to improve cognitive function in humans (Hallschmid et al., 2007; Reger et al., 2008). However, recent data about a clinical trial with mild cognitive impairment (MCI) or moderate AD patients revealed no significant effects of long-term intranasal insulin delivery on cognitive performance in memory task (Craft et al., 2017).

Finally, genetic and experimental data about insulin degrading enzyme and, more recently, the Aβ metabolism regulation by sortilin related VPS10 domain containing receptor 1 (SorCS1) gene suggest novel mechanistic links between BIR and AD (Lane et al., 2010; Wang et al., 2015). Thus, BIR seems to play a pivotal role at the crossroad between metabolic and neurodegenerative diseases, independently from the cerebrovascular mechanisms.

## Biomarkers of Brain Insulin Resistance

In view of the close relationship among metabolic diseases, BIR and cognitive decline, it is emerging the need to identify biomarkers able to detect BIR before, or possibly even in the absence of, peripheral insulin resistance, that may be predictive of age- and dementia-related cognitive impairment. Ideal biomarkers should be reliable, simple to measure, non-invasive and inexpensive (Noel-Storr et al., 2013). In this regard, the dosage of both Aβ and tau proteins in the cerebrospinal fluid is invasive and most likely indicative of a pathology already under development. For these reasons, in the last years several studies focused on evaluation of brain glucose metabolism and analysis of brain-derived extracellular vesicles extracted from the blood as biomarkers of BIR and early-phase cognitive decline.

Cerebral glucose metabolism is tightly correlated with neuronal activity (Simpson et al., 2007). Therefore, imaging of local brain hypo-metabolism can be used to visualize areas of reduced synaptic activity. The most frequently used method of brain metabolic imaging is positron emission tomography (PET) with (^18^F)fluorodeoxyglucose (FDG) (Cohen and Klunk, 2014). Reduced cerebral glucose metabolism represents one of the earliest signs of AD, and studies in both humans and experimental models suggest that altered brain glucose metabolism is associated with AD progression (Kapogiannis and Mattson, 2011; Ishibashi et al., 2015).

Recent work have identified in GK rats reduced glutamine synthesis and impairment of the glutamate-glutamine cycle between astrocytes and neurons, driving to diabetes-induced neurodegeneration and cognitive dysfunction (Girault et al., 2017). In a mouse model of AD, impaired glucose transport through the BBB and decreased cerebral lactate release during neuronal activity occur at early stages of the phenotype (Merlini et al., 2011). Dysregulated brain glucose metabolism resembling changes observed in AD patients has been observed in metabolic disorders such as obesity or T2D (Tschritter et al., 2006, 2007). However, whether neuroimaging changes of brain glucose metabolism anticipate the onset of neurodegeneration or are related to the development of BIR in the same brain areas remain still poorly understood.

More recently, molecular strategies have been developed to selectively isolate brain-derived exosomes (BDE) from biological fluids (Tschritter et al., 2006, 2007; Fiandaca et al., 2015; Goetzl et al., 2016). Exosomes are extracellular vesicles carrying information (e.g., proteins, lipids, and nucleic acids) to distant cells, which are emerging as novel potential biomarkers for human diseases (Tkach and Thery, 2016). Several pathogenic proteins that are involved in neurodegenerative diseases, including AD, are loaded into vesicles and then extracellularly secreted via exosomes (Rajendran et al., 2006; Sharples et al., 2008). More importantly, changes of insulin resistance molecular markers (i.e., higher serine phosphorylation and lower tyrosine phosphorylation of IRS-1) have been found in neural-derived exosomes extracted from blood of AD patients compared to age- and gender-matched patients with frontotemporal dementia or T2D (Kapogiannis et al., 2015). These differences were detectable up to 10 years before the onset of AD symptoms. Finally, exosomal biomarkers of BIR were associated with higher brain atrophy in AD patients (Mullins et al., 2017) emphasizing the potential role of brain derived microvesicles as detectors of brain insulin signaling and biomarkers of brain damage due to metabolic and neurodegenerative disorders.

## Conclusion

Molecules involved in metabolic homeostasis are now recognized to exert a great influence on hippocampal plasticity, and alteration of their equilibrium has a strong impact at the functional and behavioral levels. Insulin exerts a trophic role into the brain and it may also act as a signal of positive metabolic homeostasis promoting neuroplasticity, which is a high energy demanding process. Insulin plays a pivotal role in the regulation of central nervous system homeostasis and higher functions such as learning and memory, by controlling both NSC fate and the activity of neuronal network. In this regard, identifying the molecular targets that underlie the effects of insulin on brain plasticity may contribute to understand the mechanisms regulating neural plasticity in health and metabolic diseases and reveal novel targets in pathologies characterized by impaired neural plasticity, especially AD.

Actually, we do not yet have an exhaustive understanding of how systemic and brain insulin resistance are related to brain aging and AD, but clinical and experimental evidence indicates that insulin supplementation can be a therapeutic tool for patients with cognitive impairment and an added value in the treatment of dementia (Chapman et al., 2018; Santiago and Hallschmid, 2019). The availability of BDE, such as other biomarkers of brain metabolism detectable in the plasma, will foster clinical studies to identify novel therapeutic approaches for personalized medicine in neurodegenerative diseases.

## Author Contributions

All authors conceived the work, took part to the scientific discussion, and wrote the manuscript.

## Conflict of Interest Statement

The authors declare that the research was conducted in the absence of any commercial or financial relationships that could be construed as a potential conflict of interest. The reviewer VT, declared a past co-authorship with one of the authors CG, and the reviewer AG, declared a shared affiliation, with no collaboration, with several of the authors, SF and CG, to the handling Editor at the time of review.
